# Universal Stress Proteins Contribute *Edwardsiella ictaluri* Virulence in Catfish

**DOI:** 10.3389/fmicb.2018.02931

**Published:** 2018-11-28

**Authors:** Ali Akgul, Seong Won Nho, Safak Kalindamar, Hasan C. Tekedar, Hossam Abdalhamed, Mark L. Lawrence, Attila Karsi

**Affiliations:** Department of Basic Sciences, College of Veterinary Medicine, Mississippi State University, Starkville, MS, United States

**Keywords:** stress, ESC, USP, mutation, vaccine

## Abstract

*Edwardsiella ictaluri* is an intracellular Gram-negative facultative pathogen causing enteric septicemia of catfish (ESC), a common disease resulting in substantial economic losses in the U.S. catfish industry. Previously, we demonstrated that several universal stress proteins (USPs) are highly expressed under *in vitro* and *in vivo* stress conditions, indicating their importance for *E. ictaluri* survival. However, the roles of these USPs in *E. ictaluri* virulence is not known yet. In this work, 10 *usp* genes of *E. ictaluri* were in-frame deleted and characterized *in vitro* and *in vivo*. Results show that all USP mutants were sensitive to acidic condition (pH 5.5), and *Ei*Δ*usp05* and *Ei*Δ*usp08* were very sensitive to oxidative stress (0.1% H_2_O_2_). Virulence studies indicated that *Ei*Δ*usp05*, *Ei*Δ*usp07*, *Ei*Δ*usp08*, *Ei*Δ*usp09*, *Ei*Δ*usp10*, and *Ei*Δ*usp13* were attenuated significantly compared to *E. ictaluri* wild-type (*Ei*WT; 20, 45, 20, 20, 55, and 10% vs. 74.1% mortality, respectively). Efficacy experiments showed that vaccination of catfish fingerlings with *Ei*Δ*usp05*, *Ei*Δ*usp07*, *Ei*Δ*usp08*, *Ei*Δ*usp09*, *Ei*Δ*usp10*, and *Ei*Δ*usp13* provided complete protection against *Ei*WT compared to sham-vaccinated fish (0% vs. 58.33% mortality). Our results support that USPs contribute *E. ictaluri* virulence in catfish.

## Introduction

Enteric septicemia of channel catfish (ESC) is one of the most prevalent diseases of cultured catfish, causing significant losses (USDA, 2014). The most common practice in ESC treatment is use of feed medicated with oxytetracycline, sulfadimethoxine, or florfenicol. However, one of the earliest clinical signs of ESC is reduced appetite. Thus, these antimicrobials are only useful in limiting the spread of an outbreak and rather than treating the disease. Also, medicated feed may lead to the emergence of resistant *Edwardsiella ictaluri* strains (Tu et al., 2008).

The universal stress proteins (USP) have a conserved domain of 140–160 amino acids, and are present in archaea, bacteria, and plants (Nachin et al., 2005), but not in animals and human (Siegele, 2005). In *Escherichia coli usp* are involved in various functions from oxidative stress to adhesion and motility (Nachin et al., 2005). Under stress, USPs are overproduced and through a variety of mechanisms aid the survival of organism in stressful conditions (Heermann et al., 2009b). The *uspA* mutation caused decreased survival in *E. coli* (Tkaczuk et al., 2013). It is known that USPs are needed by pathogens (Hensel, 2009). USPs affect persistence and survival of *Mycobacterium tuberculosis* (Hingley-Wilson et al., 2010), and cause growth arrest and reduce the virulence in *Salmonella typhimurium* C5 (Liu et al., 2007) and *Burkholderia pseudomallei* (Al-Maleki et al., 2014). USPs are also necessary for the intracellular growth adaption of *Listeria monocytogenes* (Chatterjee et al., 2006). Similarly, *Staphylococcus aureus* virulence factors were downregulated *in vivo* while expression of *uspA* increased (Chaffin et al., 2012). *Acinetobacter baumannii uspA* is essential in pneumonia and pathogenesis (Elhosseiny et al., 2015).

Although increased expression of several *usp* genes in *E. ictaluri* under various stressors has been reported (Akgul et al., 2018), the role of USPs in *E. ictaluri* virulence is not known yet. Therefore, in this study, 10 *E. ictaluri usp* genes were studied by introducing in-frame deletions and determining their survival under acidic and oxidative stress conditions. Also, the virulence and protective properties of mutants against ESC infection were tested in catfish fingerlings.

## Materials and Methods

### Animals

All fish experiments were performed based on a protocol approved by the Mississippi State University Institutional Animal Care and Use Committee (protocol number 15-043). Channel catfish fingerlings were obtained from the fish hatchery at the College of Veterinary Medicine, Mississippi State University, and maintained at 25–28°C during experiments. Tricaine methanesulfonate (MS-222, Western, Chemical, Inc.) was used to sedate (100 mg/ml) or euthanize (400 mg/ml) the catfish.

### Bacterial Strains, Plasmids, and Growth Conditions

Bacterial strains and plasmids used in this work are listed in Table 1. *E. ictaluri* 93–146 wild-type (WT) was grown at 30°C using Brain Heart Infusion (BHI) broth and agar (Difco, Sparks, MD, United States). *E. coli* strains were cultured at 37°C using Luria-Bertani (LB) broth and agar (Difco). *E. coli* CC118λ*pir* was used for cloning and SM10λ*pir* or BW19851 were used for transferring pMEG-375 or pAK*gfplux*1 into *E. ictaluri.* When required, the following antibiotics and reagents (Sigma-Aldrich, Saint Louis, MN, United States) were added to culture medium at the following concentrations: ampicillin (Amp: 100 μg/ml), colistin (Col: 12.5 μg/ml), sucrose (5%), and mannitol (0.35%).

**Table 1:** Bacterial strains and plasmids used in this study.

Strain	Relevant characteristics	Reference
***Edwardsiella ictaluri***		
93–146	Wild type; pEI1+; pEI2+; Col^r^	Lawrence et al., 1997
*Ei*Δ*usp02*	93–146 derivative; pEI1+; pEI2+; Colr; Δ*usp02*	This study
*Ei*Δ*usp03*	93–146 derivative; pEI1+; pEI2+; Colr; Δ*usp03*	This study
*Ei*Δ*usp04*	93–146 derivative; pEI1+; pEI2+; Colr; Δ*usp04*	This study
*Ei*Δ*usp05*	93–146 derivative; pEI1+; pEI2+; Colr; Δ*usp05*	This study
*Ei*Δ*usp06*	93–146 derivative; pEI1+; pEI2+; Colr; Δ*usp06*	This study
*Ei*Δ*usp07*	93–146 derivative; pEI1+; pEI2+; Colr; Δ*usp07*	This study
*Ei*Δ*usp08*	93–146 derivative; pEI1+; pEI2+; Colr; Δ*usp08*	This study
*Ei*Δ*usp09*	93–146 derivative; pEI1+; pEI2+; Colr; Δ*usp09*	This study
*Ei*Δ*usp10*	93–146 derivative; pEI1+; pEI2+; Colr; Δ*usp10*	This study
*Ei*Δ*usp13*	93–146 derivative; pEI1+; pEI2+; Colr; Δ*usp13*	This study
***Escherichia coli***		
CC118λ*pir*	D(ara-leu); *araD*; DlacX74; *galE*; *galK*; *phoA20*; *thi-1*; *rpsE*; *rpoB*; *argE*(Am); *recAl*; lpirR6K	Herrero et al., 1990
SM10λ*pir*	*thi; thr; leu; tonA; lacY; supE; recA*;::RP4-2-Tc::Mu; Km^r^; lpirR6K	Miller and Mekalanos, 1988
BW19851λ*pir*	RP4-2 (Km::Tn7, Tc::Mu-1), DuidA3::pir+, recA1, endA1, thi-1, hsdR17, creC510	Metcalf et al., 1994
**Plasmids**		
pMEG-375	8142 bp, Amp^r^, Cm^r^, lacZ, R6K ori, *mob incP, sacR sacB*	Dozois et al., 2003
pAK*gfplux*1	5681 bp, PstI, EcoRI, HpaI, AseI, BstBI	Karsi et al., 2006
p*Ei*Δ*usp02*	9939 bp, Δ*usp02*, pMEG-375	This study
p*Ei*Δ*usp03*	9960 bp, Δ*usp03*, pMEG-375	This study
p*Ei*Δ*usp04*	10096 bp, Δ*usp04*, pMEG-375	This study
p*Ei*Δ*usp05*	10080 bp, Δ*usp05*, pMEG-375	This study
p*Ei*Δ*usp06*	10101 bp, Δ*usp06*, pMEG-375	This study
p*Ei*Δ*usp07*	10026 bp, Δ*usp07*, pMEG-375	This study
p*Ei*Δ*usp08*	10087 bp, Δ*usp08*, pMEG-375	This study
p*Ei*Δ*usp09*	9843 bp, Δ*usp09*, pMEG-375	This study
p*Ei*Δ*usp10*	9795 bp, Δ*usp10*, pMEG-375	This study
p*Ei*Δ*usp13*	9975 bp, Δ*usp13*, pMEG-375	This study

### Construction of In-Frame Deletion Mutants

The nucleotide sequences of 10 *E. ictaluri usp* genes were obtained from the *E. ictaluri* 93–146 genome (GenBank accession: CP001600), and four primers were designed for each gene (Tables 2, 3). Restriction sites were included in forward and reverse primers. Overlap extension PCR was used to delete the functional *usp* genes from the *E. ictaluri* genome (Horton et al., 1990). Genomic DNA was isolated from *E. ictaluri* using a DNeasy Blood & Tissue Kit (Qiagen, Valencia, CA, United States) and used as template in PCR. The upstream and downstream regions of each gene were amplified, and products were gel-extracted using a QIAquick Gel Extraction Kit (Qiagen). The amplified upstream and downstream fragments were mixed equally and used as a template in the subsequent overlap extension PCR to generate the in-frame deletion fragment for each gene. The in-frame deletion fragments were digested with appropriate restriction enzymes (NEB) (Table 2) and cleaned up. The suicide plasmid pMEG-375 was purified from an overnight *E. coli* culture by a QIAprep Spin Miniprep Kit (Qiagen) and digested with appropriate restriction enzymes respective to the inserts. The in-frame deletion fragments were ligated into the linearized pMEG-375 vector using T4 DNA Ligase (NEB) at 16°C overnight. *E. coli* CC118λ*pir* was transformed by electroporation and plated on LB agar plus ampicillin. Resulting plasmids were isolated from the colonies and confirmed by size, restriction enzyme digestion, and finally by sequencing. The resulting plasmids named as p*Ei*Δ*usp02*-*10* and p*Ei*Δ*usp13* were transferred into *E. coli* SM10λ*pir* or BW19851 by chemical transformation and mobilized into *E. ictaluri* WT by conjugation. First integration was selected by ampicillin, and ampicillin resistant colonies were propagated on BHI agar to allow for the second crossover allelic exchange. After this step, colonies were streaked on counter selective BHI plates with 5% sucrose, 0.35% mannitol, and colistin to allow loss of pMEG-375. Potential mutant colonies were tested for ampicillin sensitivity to ensure the loss of the plasmid, confirmed by PCR, and sequencing.

**Table 2:** The primers used for mutant construction and sequence validation.

Genes	Primer ID		Primer Sequence (5′-3′)	RE
*Eiusp*02	EI1751EF01	A	cccc**tctaga**agtgcggattgcattaca	*Xba*I
	EI1751IR01	B	gaggtcgatggaaaccag	
	EI1751IF01	C	ctggtttccatcgacctcgtactggtcgtccgctgatc	
	EI1751ER01	D	cccc**ggatcc**gattaacaacggcaaagtgg	*BamH*I
	1751F		acctgtgccatttccgctgcc	
*Eiusp*03	EI1786EF01	A	cccc**gcggccgc**ttttcgtcgcgatagacttc	*Not*I
	EI1786IR01	B	gacgggaaccaaaatcgtc	
	EI1786IF01	C	gacgattttggttcccgtcaccaccagcgtcttggtagtg	
	EI1786ER01	D	cccc**gagctc**cagctgctccatgaaattacg	*Sac*I
	1786F		gtatggcggtgataacatcc	
*Eiusp*04	EI1962EF01	A	cccc**gcggccg**cggaaaaacgtgtcattcgtc	*Not*I
	EI1962IR01	B	gttttggttcggatcgatag	
	EI1962IF01	C	ctatcgatccgaaccaaaacgaagatgagcatgatgac	
	EI1962ER01	D	cccc**gcatgc**catctctttcctgctgatgc	*Sph*I
	1962F		tgatttgttgctcgtcggta	
*Eiusp*05	EI1981EF01	A	cccc**gcggccg**cggatcatatagcccatgctg	*Not*I
	EI1981IR01	B	ggatccggttttaagatcaag	
	EI1981IF01	C	cttgatcttaaaaccggatccgacaccattagcattgatacg	
	EI1981ER01	D	cccc**gagctc**gaaatcctgacagccacttctg	*Sac*I
	1981F		ttaccatggcgcatttaggc	
*Eiusp*06	EI2616EF01	A	cccc**gcggccg**cattgtgacggaggagagatg	*Not*I
	EI2616IR01	B	cagaaccagaacatggtg	
	EI2616IF01	C	caccatgttctggttctggaccttgagaccgacgttctgg	
	EI2616ER01	D	cccc**gagctc**tgggaaatggtaaaacatgg	*Sac*I
	2616F		atatccgtcgccgtcatacc	
*Eiusp*07	EI2891EF01	A	cccc**gcggccg**cgcgctgatcatcgtcttactg	*Not*I
	EI2891IR01	B	ctgtgccagcagggtgtc	
	EI2891IF01	C	gacaccctgctggcacaggaaaccgataaggagatgacagac	
	EI2891ER01	D	cccc**gcatgc**gatacaggagcagggagttctgg	*Sph*I
	2891F		cgtcgaggctctgattacca	
*Eiusp*08	EI3729EF01	A	cccc**gcggccg**ctctccgacctgtaacaatcc	*Not*I
	EI3729IR01	B	cggagaaaggtctacagcaac	
	EI3729IF01	C	gttgctgtagacctttctccgcatatcgacatgctgatcgtc	
	EI3729ER01	D	cccc**gagctc**agcagcttgccatagttcag	*Sac*I
	3729F		gcgtttacaactgactccgg	
*Eiusp*09	EI3778EF01	A	cccc**gcggccg**caatcggtgtagaaggtgtcg	*Not*I
	EI3778IR01	B	ctcttcaatatcgacaggtac	
	EI3778IF01	C	gtacctgtcgatattgaagagaagaccaatgtgctggtg	
	EI3778ER01	D	cccc**gagctc**agaatcagggaggagtccag	*Sac*I
	3778F		acaatctccggactctgtgg	
*Eiusp*10	Ei1634EF01	A	at**cccggg**tatttgctacccctacagtgcc	*Xma*I
	Ei1634IR01	B	cagatcgaggagtgtactcat	
	Ei1634IF01	C	atgagtacactcctcgatctggatcagccgacacaaagcctc	
	Ei1634ER01	D	at**gcatgc**cgacggtgttggatgagagct	*Sph*I
	1634F		ccaccgaacacactagcaata	
*Eiusp*13	Ei3810EF01	A	at**cccggg**agcatcagtaccaccatcag	*Xma*I
	Ei3810IR01	B	ggtcagggttgcagtcttatg	
	Ei3810IF01	C	cataagactgcaaccctgacccagttaaacgcacgctatcag	
	Ei3810ER01	D	at**tctaga**cggacaatgcggatgatctga	*Xba*I
	3810F		tcagctgtgtgggtagactg	

*Primers A, B, C, and D were used for mutant construction. Bold letters show restriction enzymes added to A and D primers. Underlined letters in primer C indicate reverse complemented primer B sequence. The last primer in each group used for sequence confirmation.*

**Table 3:** Summary of *E. ictaluri usp* genes and in-frame deletion.

Gene	Locus ID	Gene ID	ORF size (bp)	Remaining US ORF (bp)	Remaining DS ORF (bp)	Deleted ORF (bp)/(%)
*usp02*	NT01EI_1751	*uspF*	435	15	0	420/(97)
*usp03*	NT01EI_1786	–	432	8	6	418/(97)
*usp04*	NT01EI_1962	*uspE*	960	28	6	926/(96)
*usp05*	NT01EI_1981	*uspA*	417	24	21	372/(89)
*usp06*	NT01EI_2616	*uspA*	420	12	14	394/(94)
*usp07*	NT01EI_2891	*kdpD*	2709	30	9	2670/(99)
*usp08*	NT01EI_3729	*uspA*	438	12	27	399/(91)
*usp09*	NT01EI_3778	*uspA*	429	18	12	399/(93)
*usp10*	NT01EI_1634	–	258	0	9	249/(97)
*usp13*	NT01EI_3810	*cpxP*	480	3	63	415/(86)

*US, Upstream; DS, Downstream; ORF, Open reading frame.*

### Construction of Bioluminescent USP Mutants

The constructed USP mutants were made bioluminescence using pAK*gfplux*1 plasmid as described previously (Karsi and Lawrence, 2007). Briefly, the overnight culture of both recipient (USP mutants) and donor cells (*E. coli* SM10λ*pir* carrying pAK*gfplux*1) were mixed at 1:2 ratio (donor : recipient) and centrifuged briefly. Pellet was transferred onto sterile 0.45 μM filter papers placed on a BHI agar and incubated at 30°C for 24 h. Bacteria on the filter paper were collected in BHI broth with ampicillin and colistin and then spread on BHI plates containing ampicillin and colistin. After incubation at 30°C for 24–48 h, ampicillin resistant bioluminescent *E. ictaluri* colonies carrying pAK*gfplux*1 appeared on plates.

### Growth Kinetics of the *E. ictaluri* USP Mutants in BHI

Growth kinetics of the ten *E. ictaluri* USP mutants was compared to *E. ictaluri* WT in BHI medium as previously described (Abdelhamed et al., 2016). Each bacterial strain had four replicates. Overnight cultures were grown in a shaking incubator at 30°C for 18 h. The optical densities (OD_600_) were measured, and adjusted volumes were added to 15 ml fresh BHI (1:100 dilution). Cultures were grown for 24 h by sampling and measuring OD_600_ values at 2, 4, 8, 12, and 20 h.

### Survival of *E. ictaluri* USP Mutants in Low pH Stress

Survival of bioluminescent USP mutants and *Ei*WT was determined under acidic stress (pH 5.5) as previously described (Seifart Gomes et al., 2011). Bacteria were cultured overnight, and OD_600_ values were used to adjust culture volumes. The experiment was performed in 96 well black plates with four replicates at acidic and neutral pH. For each well, 5 μl of bacteria were inoculated into 195 μl of BHI broth plus ampicillin and colistin. The plates were incubated in Cytation 5 Cell Imaging Multi-Mode Reader (BioTek, Winooski, VT, United States), and the photon emissions were collected for 3 h at 30°C. Bioluminescence imaging (BLI) of the 96-well plate was taken using IVIS 100 Series (Caliper Corporation, Hopkinton, MA, United States). Three independent experiments were done and used for statistical analysis.

### Survival of *E. ictaluri* USP Mutants in Oxidative Stress

The survival of the ten USP mutants in BHI supplemented with 0.1% of H_2_O_2_ were determined as previously described (Seifart Gomes et al., 2011). The experiment was performed in 96 well plates with four replicates under oxidative stress and normal conditions. The plates were incubated in Cytation 5 Cell Imaging Multi-Mode Reader, and the photon emissions were collected for 3 h at 30°C.

### Virulence and Efficacy of *E. ictaluri* USP Mutants in Catfish Fingerlings

Virulence and vaccine efficacy trials were conducted as reported by our group (Karsi et al., 2009). Approximately 720 channel catfish fingerlings (average: 13.728 cm, 10.544 g) were stocked into 36 tanks at a rate of 20 fish/tank. Tanks were divided into twelve groups with three replicate tanks each group. The experiment included 10 *E. ictaluri* USP mutants, positive control (*Ei*WT), and negative control (BHI exposed). After 1 week of acclimation, fish were challenged/vaccinated by immersion with 1.3 × 10^7^ CFU/ml water for 1 h. Mortalities were recorded daily for 21 days, and the mean percent mortalities were calculated for each treatment group. Protective properties of USP mutants against *Ei*WT infection was determined by challenging vaccinated catfish with *Ei*WT (2.8 × 10^7^ CFU/ml water). Fish mortalities were recorded daily, and the percent mortality was calculated for each group.

### Statistical Analysis

For the growth kinetic experiment, significant differences between *Ei*WT and USP mutants were determined by Student’s *t*-test. For acid and hydrogen peroxide assays, photon counts were log_10_ transformed *t*-tests were conducted. Percent reduction in bioluminescence was calculated by dividing mean photon emissions of USPs to mean photon emission of *Ei*WT. For fish experiments, percent mortalities were arcsine transformed, and analysis of variance (ANOVA) was carried out using PROC GLM of SAS v9.4 (SAS Institute, Inc., Cary, NC, United States). In virulence/vaccination trial, the percent mortalities of USP mutants were compared to that of *Ei*WT, while in efficacy trail, the comparisons were made to the sham-vaccinated group at the alpha level of 0.05.

## Results

### Construction of the *E. ictaluri* USP Mutants

**FIGURE 1:**
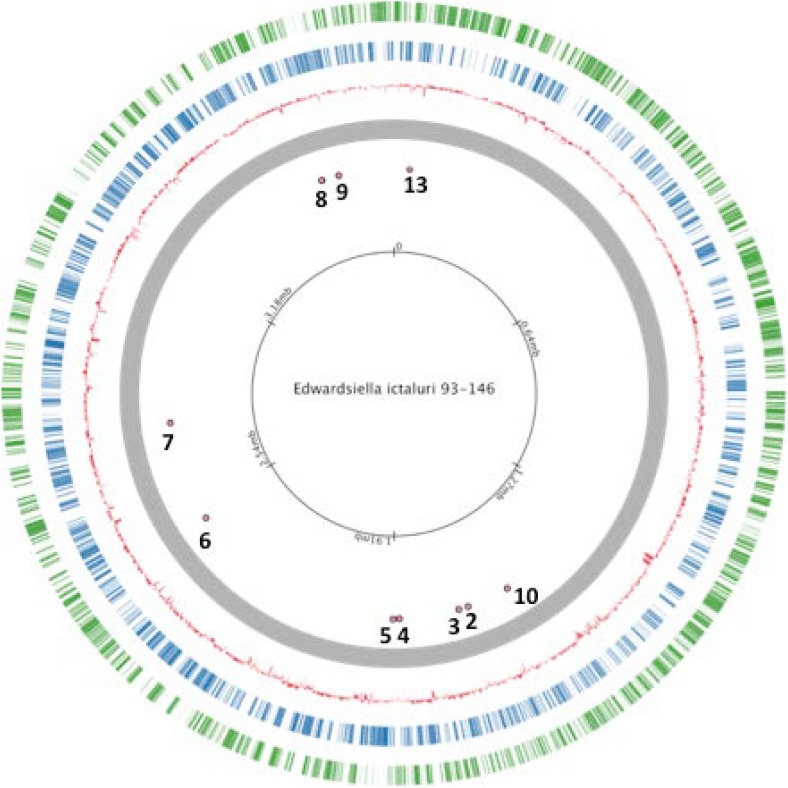
Locations of studied universal stress proteins in *Edwardsiella ictaluri* strain 93–146 genome.

**FIGURE 2:**
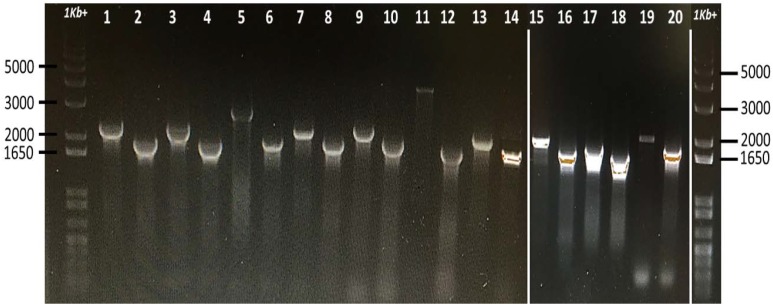
Confirmation of *E. ictaluri* USP mutants by using forward (A) and reverse (D) primers. Fragments were amplified from mutant and wild-type strains and separated on two different 1% agarose gels, which were then combined (white lines above indicate joints). A 1 Kb+ marker lane was also added to the end. Lane 1, *Ei*WT (*usp02*) and lane 2, *Ei*Δ*usp02*; lane 3, *Ei*WT (*usp03*) and lane 4, *Ei*Δ*usp03*; lane 5, *Ei*WT (*usp*04) and lane 6, *Ei*Δ*usp04*; lane 7 is *Ei*WT (*usp05*) and lane 8, *Ei*Δ*usp05*; lane 9 is *Ei*WT *(usp06)* and lane 10 *is Ei*Δ*usp06*; lane 11 is *Ei*WT (*usp07*) and lane 12 is *Ei*Δ*usp07*; lane 13 is *Ei*WT (*usp08*) and lane 14 is *Ei*Δ*usp08*; lane 15 is *Ei*WT (*usp09*) and lane 16 is *Ei*Δ*usp0*9; lane 17 is *Ei*WT *(usp10)* and lane 18 is *Ei*Δ*usp10*; lane 19 is *Ei*WT (*usp13*) and lane 20 *is Ei*Δ*usp13*.

Thirteen universal stress proteins were identified in the *E. ictaluri* genome (Williams et al., 2012) by sequence similarity (Figure 1). They were scattered through the chromosome, and no operon structure was observed. We were able to delete 10 *E. ictaluri usp* genes in-frame, and mutants were verified by PCR (Figure 2) as well as sequencing. Properties of wild-type and mutated *usp* genes are shown in Table 3. In-frame deletion resulted in removal of a large portion (86–99%) of the wild-type *usp* genes (Table 3).

### Growth Kinetics of the *E. ictaluri* USP Mutants in BHI

**FIGURE 3:**
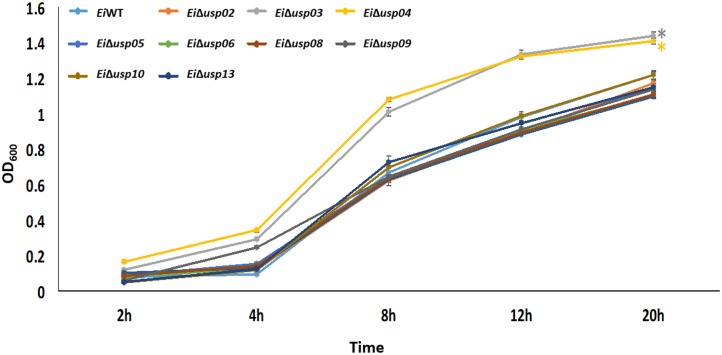
Growth of *E. ictaluri* USP mutants and WT in BHI broth. The data represent means of four replicates. *Ei*Δ*usp03* and *Ei*Δ*usp04* have a significantly (*p* < 0.001) higher growth rate than *Ei*WT and other USP mutants, which indicated by a “^∗^.” No significant differences were observed in the growth kinetics of *Ei*WT and *Ei*Δ*usp02*, *Ei*Δ*usp05*, *Ei*Δ*usp0*6, *Ei*Δ*usp07*, *E*iΔ*usp08*, *Ei*Δ*usp09*, *Ei*Δ*usp10*, and *Ei*Δ*usp13* strains.

The growth of *Ei*WT and USP mutants in BHI broth indicated that *Ei*Δ*usp03* and *Ei*Δ*usp04* have a significantly (*p* < 0.001) higher growth rate than *Ei*WT. After 20 h incubation, the growth of *Ei*WT was 23.6 and 17.42% lower than *Ei*Δ*usp03* and *Ei*Δ*usp04*, respectively (Figure 3). Whereas, no significant differences were observed in the growth kinetics of *Ei*WT and *Ei*Δ*usp02*, *Ei*Δ*usp05*, *Ei*Δ*usp06*, *Ei*Δ*usp07, Ei*Δ*usp08, Ei*Δ*usp09, Ei*Δ*usp10*, and *Ei*Δ*usp13* strains at all tested time points.

### Survival of *E. ictaluri* USP Mutants in Low pH Stress

**FIGURE 4:**
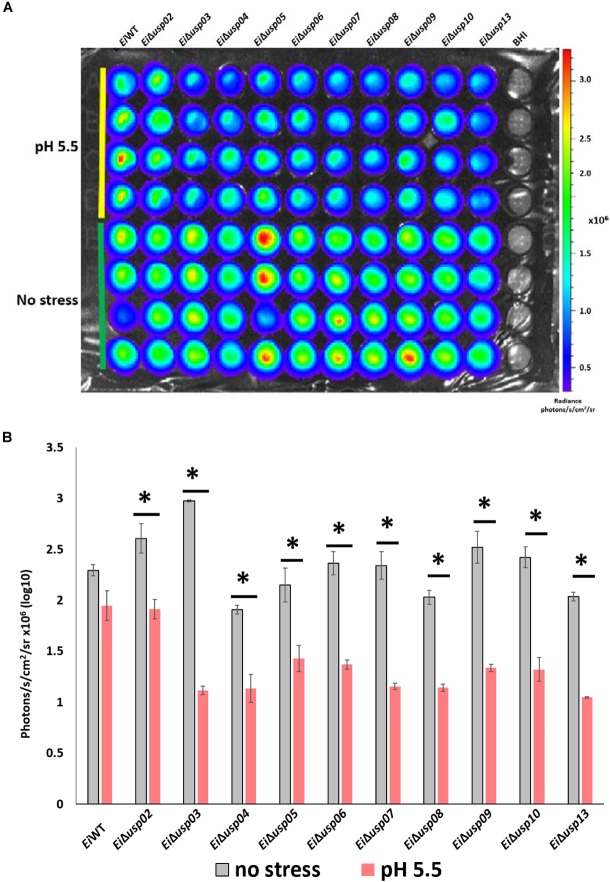
The survival assay of *E. ictaluri* WT and USP mutants in pH 5.5. **(A)** Each strain had four replicates (column A–D). Strains start with *E. ictaluri WT*, *Ei*Δ*usp02-13* and BHI control. **(B)** The bars show the difference between bioluminescence of USP mutants and WT. ^∗^indicates a significant difference between stress and non-stress at *P* < 0.01.

To evaluate the role of *usp* genes in survival and growth of *E. ictaluri* at low pH, mutants and *Ei*WT were exposed to acidic pH (5.5) and neutral pH, and bacterial growth (quantified by bioluminescent signal) were calculated. The growth rate (photon numbers) of the all USP mutants in low pH was significantly lower than that of in neutral pH. In contrast, the growth of *Ei*WT at low pH was lower but not significant (Figures 4A,B). The strongest effect of low pH was observed in *Ei*Δ*usp03* growth (62% reduction) compared to *Ei*WT. The order of susceptibility of USP mutants in low pH as follows: Δ*usp*03 > Δ*usp07* > Δ*usp13* > Δ*usp09* > Δ*usp10* > Δ*usp08* > Δ*usp06* > Δ*usp04* > Δ*usp05* > Δ*usp02*. The reduced growth of the USP mutants indicates that *usp* genes contribute *E. ictaluri* survival under acidic conditions.

### Survival of *E. ictaluri* USP Mutants in Oxidative Stress

**FIGURE 5:**
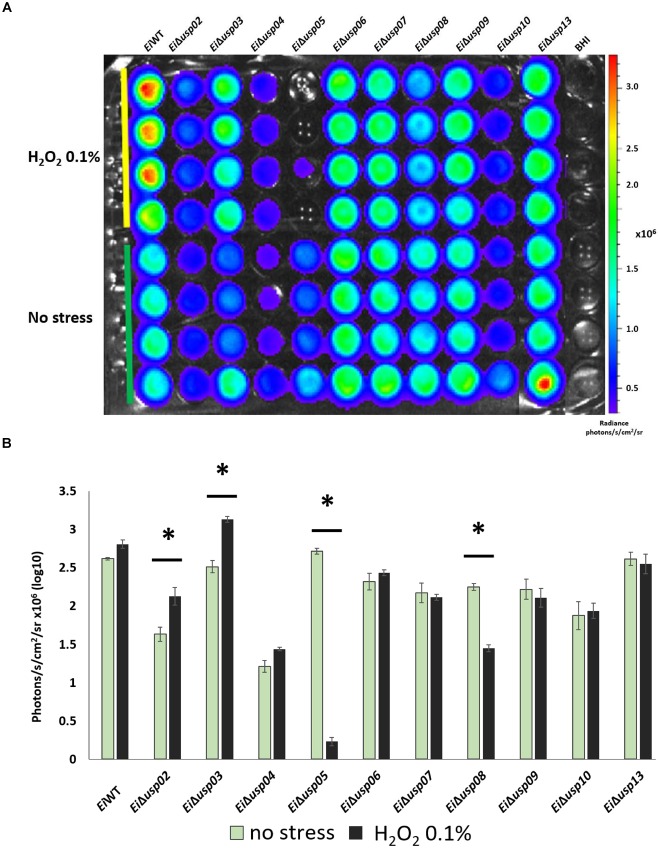
The survival assay of *E. ictaluri* WT and USP mutants exposed to 0.1% H_2_O_2_. **(A)** Each strain had four replicates (column A–D). Strains start with *E. ictaluri WT*, *Ei*Δ*usp02-13* and BHI control. **(B)** The bars show the difference between bioluminescence of USP mutants and WT. **^∗^**indicates a significant difference between stress and non-stress at *P* < 0.01.

Exposure to hydrogen peroxide (0.1% H_2_O_2_) significantly reduced growth of *Ei*Δ*usp05* and *Ei*Δ*usp08* compared to no stress group (91 and 35% reduction, respectively), while growth of *Ei*Δ*usp02* and *Ei*Δ*usp03* increased under oxidative stress (Figures 5A,B). No differences for *Ei*Δ*usp04*, *Ei*Δ*usp06*, *Ei*Δ*usp07*, *Ei*Δ*usp09, Ei*Δ*usp10*, and *Ei*Δ*usp13* strains were observed.

### Virulence and Efficacy of *E. ictaluri* USP Mutants in Catfish Fingerlings

**FIGURE 6:**
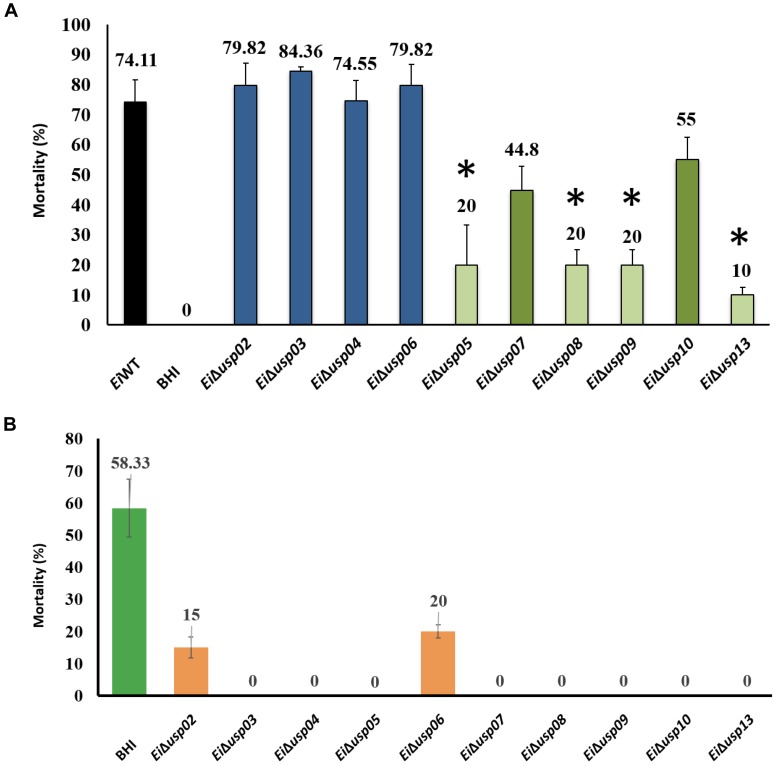
Vaccination tests of USP mutants in channel catfish fingerlings. **(A)** Percent mortalities seen after vaccination. **(B)** Percent mortalities of channel catfish fingerlings immunized with USP mutants and re-challenged with *E. ictaluri* wild-type 3 weeks post immunization. **^∗^**indicates significant differences between mutant and WT at *P* < 0.01.

The percent mortalities in catfish challenged with *Ei*Δ*usp05, Ei*Δ*usp07*, *Ei*Δ*usp08*, *Ei*Δ*usp09*, *Ei*Δ*usp10*, and *Ei*Δ*usp13* were significantly lower than that of *Ei*WT (20, 44.8, 20, 20, 55, and 10% vs. 74.1% mortality, respectively) (Figure 6A). In contrast, no significant differences between *Ei*Δ*usp02, Ei*Δ*usp03, Ei*Δ*usp04*, and *Ei*Δ*usp06* and *Ei*WT (79.8, 84.4, 74.6, and 79.82% vs. 74.1% mortality, respectively) were observed (Figure 6A). The order of attenuation in the 10 USP mutants are as following: *Ei*Δ*usp13* > *Ei*Δ*usp05* > *Ei*Δ*usp08* > *Ei*Δ*usp09* > *Ei*Δ*usp07* > *Ei*Δ*usp10* > *Ei*Δ*usp04* > *Ei*Δ*usp06* > *Ei*Δ*usp02* > *Ei*Δ*usp03*.

At 3 weeks post-immunization, *Ei*Δ*usp05, Ei*Δ*usp07*, *Ei*Δ*usp08*, *Ei*Δ*usp09*, *Ei*Δ*usp10*, and *Ei*Δ*usp13* provided significant protection against *Ei*WT challenges (no mortalities; *p* < 0.01) compared to sham-vaccinated fish (58.33% mortality) (Figure 6B). Although immunization with *Ei*Δ*usp03* and *Ei*Δ*usp04* protected catfish significantly, they were not safe. *Ei*Δ*usp05, Ei*Δ*usp08*, *Ei*Δ*usp09*, and *Ei*Δ*usp13* were both safe and protective among all USP mutants.

**FIGURE 7:**
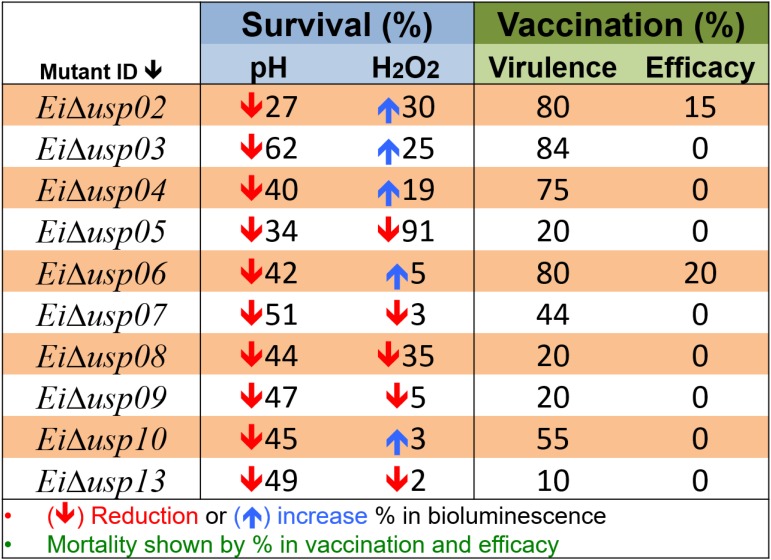
Overall summary of results. Survival percent under acidic (pH) and oxidative stress (H_2_O_2_) conditions was calculated based on changes in bioluminescence signal. The downward direction arrow indicates reduction in survival percent between mutant strain compared with wild type. The upward direction indicates increase in survival percent. Virulence percent is based on catfish mortality after immersion challenge with USP mutant strains. Efficacy perecent is based of mortality after re-challenge the immunized fish with *E. ictaluri* WT at 21 day post-immunization.

Figure 7 provides overall summary of the results.

## Discussion

Several previous studies reported that universal stress proteins (USPs) play a role in different bacteria to respond to different stress conditions, such as heat, substrate starvation, exposure to antimicrobial agents, acidic stress, and oxidative stress (Seifart Gomes et al., 2011). The objective of this study was to determine the role of *E. ictaluri usp* genes in acidic and oxidative stresses as well as in virulence. Also, mutants’ vaccine potentials were determined.

The *uspA* gene among *usp* genes has been studied in different bacterial strains. Deletion of the *uspA* genes resulted in decreased virulence in *Salmonella typhimurium* C5*, Listeria monocytogenes*, and *Acinetobacter baumannii* (Liu et al., 2007; Seifart Gomes et al., 2011; Elhosseiny et al., 2015). Also, *uspA* affected the host invasion and survival in *Salmonella enterica* and *Mycobacterium tuberculosis* (Hensel, 2009; Hingley-Wilson et al., 2010). In the present study, there were four *usp* genes (*usp05*, *usp06*, *usp08*, and *usp09*) with high similarity to *uspA*. The growth rate of *Ei*Δ*usp05, Ei*Δ*usp06, Ei*Δ*usp08*, and *Ei*Δ*usp09* were similar to *E. ictaluri* WT. However, *Ei*Δ*usp05* and *Ei*Δ*usp08* showed reduced growth in oxidative and acidic stresses compared to *Ei*WT. Virulence data showed that *Ei*Δ*usp05, Ei*Δ*usp08*, and *Ei*Δ*usp09* were significantly attenuated compared to *E. ictaluri* WT. However, *Ei*Δ*usp06* was not attenuated. These results are consistent with a previous study in *L. monocytogenes* where not all *uspA* are involved in reduced virulence (Seifart Gomes et al., 2011). Previously, our group reported that transposon insertion mutants in *usp05* reduced *E. ictaluri* virulence in catfish and provided better protection against ESC (Kalindamar, 2013). Additionally, expressions of *usp05* were very high in response to host stress or high level of H_2_O_2_ in *E. ictaluri* (Akgul et al., 2018). The *usp05* gene (*uspA)* is an important regulator of survival and virulence in many pathogens (Tkaczuk et al., 2013). In *E. coli*, *uspA* mutant caused a survival defect under a variety of growth-arrested conditions, whereas overexpression induced growth in the growth-arrested state. Our data suggest that *usp05, usp08*, and *usp09* are important virulence genes in *E. ictaluri*.

We demonstrated that *Ei*Δ*usp03* and *Ei*Δ*usp04* have a faster growth rate than *Ei*WT and other USP mutants. However, lack of *usp* genes did not cause growth differences in *Listeria monocytogenes* (Seifart Gomes et al., 2011), *E. coli* (Nystrom and Neidhardt, 1993) or other bacteria when cultured in conventional media (Liu et al., 2007; Hingley-Wilson et al., 2010). Indeed, *Ei*Δ*usp03* and *Ei*Δ*usp04* did not show any virulence attenuation in *E. ictaluri*, which was similar to USP mutant Rv2623 in *Mycobacterium tuberculosis* (Hingley-Wilson et al., 2010). This study suggested that *usp* genes might play a role in latency and persistence of chronic TB infection. We think that *usp*03 and *usp*04 are not involved in virulence but may play other roles in stress responses in *E. ictaluri*.

*Edwardsiella ictaluri* can survive and continue growth in up to 3 mM of H_2_O_2_ and low acidic pH 5.5. When the USP mutants and *Ei*WT exposed to low pH, growth rates did not change significantly. As shown previously, *L. monocytogenes* ATP Binding USPs exhibited role in the response to acid stress during exponential growth phase (Tremonte et al., 2016).

Our results indicated that *E. ictaluri usp07* contributes to virulence of *E. ictaluri*. Mortality was significantly decreased in the *Ei*Δ*usp07* mutant compared to *Ei*WT strain. The *usp*07 is a *KdpD* protein, and it contains a *uspA* domain (Heermann et al., 2009a). We included whole *KdpD* as *usp*07 because USP domain is located between the *N*-terminal sensor domain and *C*-terminal catalytic domain of this Osmo-sensitive K^+^ channel histidine kinase. Mutant *KdpD* in *Salmonella typhimurium* is attenuated in animal infection model and macrophage survival experiments. It also promotes resistance to osmotic, oxidative and antimicrobial stresses (Alegado et al., 2011). *KdpD* is also involved in oxidative-osmotic stress, response to host, and virulence (Freeman et al., 2013). In our gene expression study after host stress, *usp07* showed a very high expression level (Akgul et al., 2018). It is important to note that *usp07* involved in *E. ictaluri* virulence and acid stress response.

The *usp*13 was described as a universal stress protein and extra cytoplasmic adaptor protein (*CpxP*) like protein (Williams et al., 2012). The *usp13* (*CpxP*) is placed in the inner membrane with histidine kinase *CpxA* and *CpxR*, a response regulator (Vogt and Raivio, 2012; Debnath et al., 2013). *CpxP* is the most highly inducible member of the *Cpx* regulon, and it has elevated expression in response to both envelope stress and entry into stationary phase growth (Motohashi et al., 1999; DiGiuseppe and Silhavy, 2003). The CPX system is important and required for virulence in both Gram-negative and -positive bacteria (Raju et al., 2012). Previously, we determined that *E. ictaluri*, *usp13* is highly expressed when exposed low acidic pH (5.5) and the catfish invasion (Akgul et al., 2018). The *usp13* (*cpxP*) is an essential regulator of cell membrane stress in bacteria during host infection. Therefore, it is involved in the virulence of *E. ictaluri* with a very high reduction in virulence (Figure 6).

The expression of *E. coli usp* genes is controlled by some effector proteins and signaling molecules, such as SOS repose proteins (Gustavsson et al., 2002; Kvint et al., 2003; Persson et al., 2007). However, mechanisms of USPs in other bacterial species are not known entirely. Overall our results are in line with studies from various species that USPs were crucial for protecting the cells from the damaging effects of reactive oxygen species (ROS) (Nachin et al., 2005; Liu et al., 2007; Seifart Gomes et al., 2011; Elhosseiny et al., 2015; Figure 7).

## Conclusion

Our lab aims to develop live attenuated vaccines to protect catfish against *E. ictaluri* infections. Live attenuated bacterial should be both safe and confer full protection against wild-type infections. This study identified that *Ei*Δ*usp05*, *Ei*Δ*usp08, Ei*Δ*usp09*, and *Ei*Δ*usp13* strains have vaccine potential and further efforts, such as constructing double mutants to improve their safety, could be pursued. The data presented in this study display that USPs are essential for both stress physiology and pathogenesis in *E. ictaluri*.

## Author Contributions

AK and ML conceived the project and designed the experiments. AA, SN, SK, HT, and HA conducted the experiments. AA wrote the manuscript. SN, SK, HT, HA, ML, and AK reviewed the manuscript.

## Conflict of Interest Statement

The authors declare that the research was conducted in the absence of any commercial or financial relationships that could be construed as a potential conflict of interest.

## References

[B1] Abdelhamed H. Lu J. Lawrence M. L. Karsi A. (2016). Involvement of tolQ and tolR genes in *Edwardsiella ictaluri* virulence. *Microb. Pathog.* 100 90–94. 10.1016/j.micpath.2016.09.011 27622343

[B2] Akgul A. Akgul A. Lawrence M. L. Karsi A. (2018). Stress-related genes promote *Edwardsiella ictaluri* pathogenesis. *PLoS One* 13:e0194669. 10.1371/journal.pone.0194669 29554143 PMC5858854

[B3] Alegado R. A. Chin C. Y. Monack D. M. Tan M. W. (2011). The two-component sensor kinase KdpD is required for *Salmonella typhimurium* colonization of *Caenorhabditis elegans* and survival in macrophages. *Cell. Microbiol.* 13 1618–1637. 10.1111/j.1462-5822.2011.01645.x 21790938

[B4] Al-Maleki A. R. Mariappan V. Vellasamy K. M. Shankar E. M. Tay S. T. Vadivelu J. (2014). Enhanced intracellular survival and epithelial cell adherence abilities of *Burkholderia pseudomallei* morphotypes are dependent on differential expression of virulence-associated proteins during mid-logarithmic growth phase. *J. Proteomics* 106 205–220. 10.1016/j.jprot.2014.04.005 24742602

[B5] Chaffin D. O. Taylor D. Skerrett S. J. Rubens C. E. (2012). Changes in the *Staphylococcus aureus* transcriptome during early adaptation to the lung. *PLoS One* 7:e41329. 10.1371/journal.pone.0041329 22876285 PMC3410880

[B6] Chatterjee S. S. Hossain H. Otten S. Kuenne C. Kuchmina K. Machata S. (2006). Intracellular gene expression profile of *Listeria monocytogenes*. *Infect. Immun.* 74 1323–1338. 10.1128/IAI.74.2.1323-1338.2006 16428782 PMC1360297

[B7] Debnath I. Norton J. P. Barber A. E. Ott E. M. Dhakal B. K. Kulesus R. R. (2013). The Cpx stress response system potentiates the fitness and virulence of uropathogenic *Escherichia coli*. *Infect. Immun.* 81 1450–1459. 10.1128/IAI.01213-12 23429541 PMC3647988

[B8] DiGiuseppe P. A. Silhavy T. J. (2003). Signal detection and target gene induction by the CpxRA two-component system. *J. Bacteriol.* 185 2432–2440. 10.1128/JB.185.8.2432-2440.2003 12670966 PMC152615

[B9] Dozois C. M. Daigle F. Curtiss R. III (2003). Identification of pathogen-specific and conserved genes expressed in vivo by an avian pathogenic *Escherichia coli* strain. *Proc. Natl. Acad. Sci. U.S.A.* 100 247–252. 10.1073/pnas.232686799 12506201 PMC140941

[B10] Elhosseiny N. M. Amin M. A. Yassin A. S. Attia A. S. (2015). *Acinetobacter baumannii* universal stress protein A plays a pivotal role in stress response and is essential for pneumonia and sepsis pathogenesis. *Int. J. Med. Microbiol.* 305 114–123. 10.1016/j.ijmm.2014.11.008 25466824

[B11] Freeman Z. N. Dorus S. Waterfield N. R. (2013). The KdpD/KdpE two-component system: integrating K+ Homeostasis and virulence. *PLoS Pathog.* 9:e1003201. 10.1371/journal.ppat.1003201 23555240 PMC3610689

[B12] Gustavsson N. Diez A. Nystrom T. (2002). The universal stress protein paralogues of *Escherichia coli* are coordinately regulated and co-operate in the defence against DNA damage. *Mol. Microbiol.* 43 107–117. 10.1046/j.1365-2958.2002.02720.x 11849540

[B13] Heermann R. Lippert M.-L. Jung K. (2009a). Domain swapping reveals that the N-terminal domain of the sensor kinase KdpD in *Escherichia coli* is important for signaling. *BMC Microbiol.* 9:133. 10.1186/1471-2180-9-133 19589130 PMC2714519

[B14] Heermann R. Weber A. Mayer B. Ott M. Hauser E. Gabriel G. (2009b). The universal stress protein UspC scaffolds the KdpD/KdpE signaling cascade of *Escherichia coli* under salt stress. *J. Mol. Biol.* 386 134–148. 10.1016/j.jmb.2008.12.007 19101563

[B15] Hensel M. (2009). “Secreted proteins and virulence in *Salmonella enterica*,” in *Bacterial Secreted Proteins: Secretory Mechanisms and Role in Pathogenesis*, ed. Wooldridge K. (Nottingham: Caister Academic Press).

[B16] Herrero M. de Lorenzo V. Timmis K. N. (1990). Transposon vectors containing non-antibiotic resistance selection markers for cloning and stable chromosomal insertion of foreign genes in Gram-negative bacteria. *J. Bacteriol.* 172 6557–6567. 10.1128/jb.172.11.6557-6567.1990 2172216 PMC526845

[B17] Hingley-Wilson S. M. Lougheed K. E. Ferguson K. Leiva S. Williams H. D. (2010). Individual *Mycobacterium tuberculosis* universal stress protein homologues are dispensable in vitro. *Tuberculosis* 90 236–244. 10.1016/j.tube.2010.03.013 20541977 PMC2914252

[B18] Horton R. M. Cai Z. L. Ho S. N. Pease L. R. (1990). Gene splicing by overlap extension: tailor-made genes using the polymerase chain reaction. *Biotechniques* 8 528–535. 2357375

[B19] Kalindamar S. (2013). *Identification of the Edwardsiella Ictaluri Genes Causing Impaired Growth in Complex Medium.* Master thesis, Mississippi State University, Starkville, MS.

[B20] Karsi A. Gulsoy N. Corb E. Dumpala P. R. Lawrence M. L. (2009). High-throughput bioluminescence-based mutant screening strategy for identification of bacterial virulence genes. *Appl. Environ. Microbiol.* 75 2166–2175. 10.1128/AEM.02449-08 19201969 PMC2663204

[B21] Karsi A. Lawrence M. L. (2007). Broad host range fluorescence and bioluminescence expression vectors for gram-negative bacteria. *Plasmid* 57 286–295. 10.1016/j.plasmid.2006.11.002 17207855

[B22] Karsi A. Menanteau-Ledouble S. Lawrence M. L. (2006). Development of bioluminescent *Edwardsiella ictaluri* for noninvasive disease monitoring. *FEMS Microbiol. Lett.* 260 216–223. 10.1111/j.1574-6968.2006.00310.x 16842347

[B23] Kvint K. Nachin L. Diez A. Nyström T. (2003). The bacterial universal stress protein: function and regulation. *Curr. Opin. Microbiol.* 6 140–145. 10.1016/S1369-5274(03)00025-0 12732303

[B24] Lawrence M. L. Cooper R. K. Thune R. L. (1997). Attenuation, persistence, and vaccine potential of an *Edwardsiella ictaluri purA* mutant. *Infect. Immun.* 65 4642–4651. 9353045 10.1128/iai.65.11.4642-4651.1997 PMC175666

[B25] Liu W.-T. Karavolos M. H. Bulmer D. M. Allaoui A. Hormaeche R. D. Lee J. J. (2007). Role of the universal stress protein UspA of *Salmonella* in growth arrest, stress and virulence. *Microb. Pathog.* 42 2–10. 10.1016/j.micpath.2006.09.002 17081727

[B26] Metcalf W. W. Jiang W. Wanner B. L. (1994). Use of the rep technique for allele replacement to construct new *Eschericia coli* hosts for maintenance of R6K gamma origin plasmids at different copy numbers. *Gene* 138 1–7. 10.1016/0378-1119(94)90776-5 8125283

[B27] Miller V. L. Mekalanos J. J. (1988). A novel suicide vector and its use in construction of insertion mutations: osmoregulation of outer membrane proteins and virulence determinants in *Vibrio cholerae* requires toxR. *J. Bacteriol.* 170 2575–2583. 2836362 10.1128/jb.170.6.2575-2583.1988 PMC211174

[B28] Motohashi K. Watanabe Y. Yohda M. Yoshida M. (1999). Heat-inactivated proteins are rescued by the DnaK.J-GrpE set and ClpB chaperones. *Proc. Natl. Acad. Sci. U.S.A.* 96 7184–7189. 10.1073/pnas.96.13.7184 10377389 PMC22047

[B29] Nachin L. Nannmark U. Nystrom T. (2005). Differential roles of the universal stress proteins of *Escherichia coli* in oxidative stress resistance, adhesion, and motility. *J. Bacteriol.* 187 6265–6272. 10.1128/JB.187.18.6265-6272.2005 16159758 PMC1236625

[B30] Nystrom T. Neidhardt F. C. (1993). Isolation and properties of a mutant of *Escherichia coli* with an insertional inactivation of the uspA gene, which encodes a universal stress protein. *J. Bacteriol.* 175 3949–3956. 10.1128/jb.175.13.3949-3956.1993 8391533 PMC204822

[B31] Persson O. Valadi A. Nystrom T. Farewell A. (2007). Metabolic control of the *Escherichia coli* universal stress protein response through fructose-6-phosphate. *Mol. Microbiol.* 65 968–978. 10.1111/j.1365-2958.2007.05838.x 17640273

[B32] Raju R. M. Goldberg A. L. Rubin E. J. (2012). Bacterial proteolytic complexes as therapeutic targets. *Nat. Rev. Drug Discov.* 11 777–789. 10.1038/nrd3846 23023677

[B33] Seifart Gomes C. Izar B. Pazan F. Mohamed W. Mraheil M. A. Mukherjee K. (2011). Universal stress proteins are important for oxidative and acid stress resistance and growth of *Listeria monocytogenes* EGD-e *In vitro* and *In vivo*. *PLoS One* 6:e24965. 10.1371/journal.pone.0024965 21980369 PMC3184099

[B34] Siegele D. A. (2005). Universal stress proteins in *Escherichia coli*. *J. Bacteriol.* 187 6253–6254. 10.1128/JB.187.18.6253-6254.2005 16159755 PMC1236659

[B35] Tkaczuk K. L. Shumilin A. Chruszcz M. Evdokimova E. Savchenko A. Minor W. (2013). Structural and functional insight into the universal stress protein family. *Evol. Appl.* 6 434–449. 10.1111/eva.12057 23745136 PMC3673472

[B36] Tremonte P. Succi M. Coppola R. Sorrentino E. Tipaldi L. Picariello G. (2016). Homology-based modeling of universal stress protein from *Listeria innocua* Up-regulated under acid stress conditions. *Front. Microbiol.* 7:1998. 10.3389/fmicb.2016.01998 28066336 PMC5168468

[B37] Tu T. D. Haesebrouck F. Nguyen A. T. Sorgeloos P. Baele M. Decostere A. (2008). Antimicrobial susceptibility pattern of *Edwardsiella ictaluri* isolates from natural outbreaks of bacillary necrosis of *Pangasianodon hypophthalmus* in Vietnam. *Microb. Drug Resist.* 14 311–316. 10.1089/mdr.2008.0848 19090723

[B38] USDA (2014). *Development of Approaches to Prevent and Ameliorate Diseases of Catfish (Agricultural Research Service Annual Report).* Washington, DC: United States Department of Agriculture.

[B39] Vogt S. L. Raivio T. L. (2012). Just scratching the surface: an expanding view of the Cpx envelope stress response. *FEMS Microbiol. Lett.* 326 2–11. 10.1111/j.1574-6968.2011.02406.x 22092948

[B40] Williams M. L. Gillaspy A. F. Dyer D. W. Thune R. L. Waldbieser G. C. Schuster S. C. (2012). Genome sequence of *Edwardsiella ictaluri* 93-146, a strain associated with a natural channel catfish outbreak of enteric septicemia of catfish. *J. Bacteriol.* 194 740–741. 10.1128/JB.06522-11 22247535 PMC3264089

